# Hypoxia and HIF-1α Regulate the Activity and Expression of Na,K-ATPase Subunits in H9c2 Cardiomyoblasts

**DOI:** 10.3390/cimb45100522

**Published:** 2023-10-12

**Authors:** Beyza Gurler, Gizem Gencay, Emel Baloglu

**Affiliations:** 1 Department of Medical Biotechnology, Institute of Health Sciences, Acibadem Mehmet Ali Aydinlar University, Istanbul 34752, Turkey; beyzagurlerr@gmail.com; 2 Department of Molecular and Translational Biomedicine, Institute of Natural and Applied Sciences, Acibadem Mehmet Ali Aydinlar University, Istanbul 34752, Turkey; b.gizem.gg@gmail.com; 3 Department of Medical Pharmacology, School of Medicine, Acibadem Mehmet Ali Aydinlar University, Istanbul 34752, Turkey

**Keywords:** ischemic heart disease, hypoxia, Na,K-ATPase, HIF-1α, ouabain, H9c2 cardiomyoblasts

## Abstract

The optimal function of the Na,K-ATPase (NKA) pump is essential for the heart. In ischemic heart disease, NKA activity decreases due to the decreased expression of the pump subunits. Here, we tested whether the hypoxia-inducible transcription factor (HIF-1α), the key signaling molecule regulating the adaptation of cells to hypoxia, is involved in controlling the expression and cellular dynamics of α1- and β1-NKA isoforms and of NKA activity in in-vitro hypoxic H9c2 cardiomyoblasts. HIF-1α was silenced through adenoviral infection, and cells were kept in normoxia (19% O_2_) or hypoxia (1% O_2_) for 24 h. We investigated the mRNA and protein expression of α1-, β1-NKA using RT-qPCR and Western blot in whole-cell lysates, cell membranes, and cytoplasmic fractions after labeling the cell surface with NHS-SS-biotin and immunoprecipitation. NKA activity and intracellular ATP levels were also measured. We found that in hypoxia, silencing HIF-1α prevented the decreased mRNA expression of α1-NKA but not of β1-NKA. Hypoxia decreased the plasma membrane expression of α1-NKA and β1- NKA compared to normoxic cells. In hypoxic cells, HIF-1α silencing prevented this effect by inhibiting the internalization of α1-NKA. Total protein expression was not affected. The decreased activity of NKA in hypoxic cells was fully prevented by silencing HIF-1α independent of cellular ATP levels. This study is the first to show that in hypoxic H9c2 cardiomyoblasts, HIF-1α controls the internalization and membrane insertion of α1-NKA subunit and of NKA activity. The mechanism behind this regulation needs further investigation.

## 1. Introduction

The Na,K-ATPase (NKA) enzyme or pump first described by Skou et al. is a member of the P-type ATPase family that actively transports a variety of cations across cell membranes. NKA is ubiquitously expressed in all mammalian cells and tissues [1]. By using the energy obtained from the hydrolysis of ATP, it transports 3Na^+^ ions out of the cell and 2K^+^ ions into the cell. In the heart muscle, NKA activity is required for maintaining Na^+^ and Ca^2+^ ion gradients, excitability, propagation of action potentials, electro-mechanical coupling, trans-membrane Na^+^, K^+^ and Ca^2+^ gradients, contractility, intracellular pH, glucose, and amino acid transport into the cells [2]. Thus, the dysfunction of NKA may have detrimental effects on cardiac muscle function. NKA is also the receptor for cardiac glycosides, which are used as positive inotropic agents in the treatment of end-stage congestive heart failure in some patients. In addition to the well-known functions of NKA, studies by the group of Xie at al. demonstrated that NKA is also a signaling molecule and mediates signal transduction by tyrosine kinase Src, epidermal growth factor receptor, reactive oxygen species (ROS), Ras and p42/44 mitogen-activated protein kinases, and Ca^2+^ [3,4,5,6,7,8,9,10,11,12,13].

NKA is a hetero-oligomeric protein, and it consists of a catalytic α subunit, a regulatory β subunit, and a γ subunit, which is a member of the FXYD proteins involved in regulating the function of NKA [2,14]. The α-NKA has binding sites for Na^+^, K^+^, Mg^2+^, ATP, and cardiac glycosides.

Four isoforms of α-NKA (α1, α2, α3, and α4) are expressed at different levels depending on the tissues and species [15]. Alpha1 NKA is the ubiquitous isoform and is expressed in all cell-types; α2- and α3- isoforms are expressed mostly in the heart, skeletal muscle, and neurons. The α4- isoform is found in the testes and regulates sperm fertility [16]. In human ventricular myocardium, α1-, α2-, and α3- isoforms are expressed; in mouse and rat myocardium, α1- and α2- isoforms are expressed [17,18]. In ventricular myocytes, α1-isoform is the major and house-keeping isoform [19], which is located densely and diffusely in the plasma membrane, whereas α2- and α3- isoforms are mostly found in T-tubules [17]. The β-subunit is a single transmembrane protein with three isoforms (β1, β2, and β3). In the heart muscle, β1-isoform dominates over others, and it is required for the functionality of NKA by regulating the correct orientation and membrane stabilization of the α-isoform [20,21]. The functional determinant of the pump has been attributed to αβFXYD combination with a 1:1:1 stoichiometry [22].

A mismatch between the demand and supply of oxygen and nutrients leads to hypoxia and ischemia of the myocardium. Myocardial hypoxia is a common finding in myocardial infarction, pulmonary and systemic hypertension, and hypertrophic heart failure [23]. Under hypoxic conditions, hypoxia-inducible transcription factors (HIFs) play key roles in the adjustment of cells to low oxygen levels [24]. Cellular responses to hypoxia vary depending on the tissue, degree, and duration of hypoxia. HIF levels increase in myocardial ventricular-biopsy specimens in patients undergoing coronary bypass surgery [25] and in animal models of myocardial infarction [26,27], cardiac hypertrophy [28,29], arrhythmia [27], and pulmonary hypertension [30].

NKA activity decreases in necropsy materials from ischemic heart and animal models of heart disease [31,32,33,34,35], which causes elevated intracellular Na^+^, increased activities of Na^+^/H^+^ and Na^+^/Ca^2+^ exchangers, intracellular Ca^2+^accumulation, deterioration of diastolic function, arrhythmias, and compromised cell metabolism [36,37,38,39]. In patient tissues with heart failure and in animal models of ischemic heart disease, NKA activity decreased by 30–43% [32,33,37]. The decreased activity of NKA has been attributed to decrease in the mRNA and protein expression of the α1-, α2-, and β1- isoforms [32,34,37,40]. Some studies that used tissues from human heart failure reported decreased protein expression of α1-, α3-, and β1- NKA [32,34]; some reported no changes in α1- and β1- mRNA and protein levels [33,41,42]; while some others showed increased expression of α2- and α3- isoforms [32,42,43]. Discrepant findings also exist in tissues from various animal models. Semb et al. showed the decreased capacity of NKA without any changes in the mRNA and protein expressions of α1- and β1-NKA in a cardiac hypertrophy model; another study reported decreased α1- and α2- isoforms [33,34]. In hypertrophied myocardium of renovascular hypertension in rats, Book et al. reported no changes in α1-isoform expression but decreased β1-isoform [44]. Fedorova et al. showed increased expression of α1- isoform in a model of left ventricular hypertrophy while NKA activity decreased. In the same model, during the transition to heart failure, the abundance of α1- isoform decreased, while α3- isoform increased along with changes in the sensitivity to cardiotonic steroids [43]. Collectively, these expression studies do not clarify the reported decreased activity of NKA, and the inconsistent findings do not link to impaired pump activity.

A common condition in these models and ischemic heart disease is the hypoxia of the myocardium. It is yet unknown whether tissue hypoxia and HIF-1α is involved in regulating the expression and activity of NKA [23]. The aim of this study is to investigate the expression of α1-and β1-NKA and activity of NKA in a precisely controlled in-vitro hypoxic environment using rat ventricular H9c2 cardiomyoblasts as a model and to test the involvement of HIF-1α. Here, we report for the first time that hypoxia decreases the activity of NKA and the plasma membrane expression of α1-NKA in a HIF-1α dependent manner. 

## 2. Materials and Methods

### 2.1. Cell Culture

H9c2 cells (rat ventricular cardiomyoblasts) (ATCC, Manassas, VA, USA) were used for the study. Cells were cultured according to ATCC directions with complete DMEM (c-DMEM, Gibco™, #31885023, Paisley, UK), supplemented with 100U Penicillin–Streptomycin (Sigma, St. Louis, MO, USA, #P4433), 5 mM HEPES (Capricorn, Ebsdorfergrund, Germany), and 10% fetal bovine serum (FBS, Capricorn), in an incubator at 5% CO_2_ at 37 °C. The medium was replaced every two days, and cells were grown until confluency reached 80%. For the experiments, cells were seeded at a density of 2 × 10^5^/cm^2^. Twenty-four after-seeding cells were infected with adenoviral vectors based on cell seeding density.

### 2.2. HIF-1α Silencing Experiments and In Vitro Hypoxia

Adenoviral vectors containing shRNA sequences to silence rat HIF-1α (shHIF-1α), and a scrambled sequence (scr.co.) serving as control, has been generated and previously reported [45]. H9c2 cells were infected with 100 MOI (multiplicity of infection) in c-DMEM without antibiotics for 16 h. Then, the medium was replaced with c-DMEM. For invitro hypoxia experiments, cells were kept in an incubator in 1% O_2_ + 5% CO_2_, residual N_2_ gas at 37 °C, for 24 h (Binder, Tuttlingen, Germany). Normoxic cells were kept in 19% O_2_. The silencing efficiency of HIF-1α was tested on the mRNA level and protein expression in nuclear extracts.

### 2.3. RNA Isolation and Quantitative RT-PCR

H9c2 cells were washed with phosphate-buffered saline (PBS) and were lysed using the RLT reagent (Qiagen, Hilden, Germany). Total RNA was isolated with the RNeasy micro kit that included DNase digestion step (Qiagen, Hilden, Germany) according to the manufacturer’s instructions. RNA was transcribed with SensiFAST cDNA Synthesis Kit (#BIO-65054, Bioline, London, UK), and real-time quantitative-PCR was performed using the SensiFAST SYBR No-ROX (Bioline) and QuantiTect^®^ primers according to manufacturer’s instructions (QuantiTect^®^, Qiagen; the sequences are not available due to company’s policy). Results were analyzed using the delta-deltaCT method [46], with 28SrRNA as a reference gene, and represented as normalized values of normoxic scrambled control treated cells.

### 2.4. Preparation of Nuclear Extracts

Under hypoxic conditions, stabilized HIF-1α translocates into cell nucleus and binds to hypoxia response elements in the promoter of some genes. To check the silencing efficiency of HIF-1α, we measured protein expression in nuclear extracts from normoxic, hypoxic, or HIF-1α silenced cells. The cells were washed with ice-cold PBS; lysed with a buffer composed of (in mM) 150 NaCl, 50 Tris (pH:7.5), 5 EDTA, 0.5% NP-40, 1% Triton-X100, 1 DTT, 10 NaF, 1 NaVO_3_, and 1× protease inhibitor cocktail (Roche, #11836170001,Mannheim, Germany); incubated for 15 min at 4 °C; and centrifuged at 12,000× *g* for 1 min at 4 °C. Pellets were washed briefly with PBS, and nuclei were lysed with a buffer composed of (in mM) 300 NaCl, 50 KCl, 50 Hepes-KOH (pH: 7.9), 0.1 EDTA, 10% Glycerol, 1 DTT, 10 NaF, 1 NaVO_3_, and 1× protease inhibitor cocktail by incubating them for 30 min at 4 °C and vortexing repeatedly. Lysates were collected after centrifugation at 12,000× *g* for 20 min at 4 °C and were aliquoted and immediately frozen at −80 °C. Protein levels were measured using Bradford reagent (Bio-Rad Protein Assay Kit II).

### 2.5. Cell Surface Biotinylation Experiments

The cell surface expression of α1-and β1-NKA was measured after biotinylation of plasma membrane proteins using cell-impermeable EZ-Link Sulfo-NHS-SS-Biotin (Thermo Scientific, #21331, Rockford, IL, USA), followed by immunoprecipitation and Western blot. Cells were washed three times with ice-cold PBS; incubated with 1.25 μg/mL of Sulfo-NHS-SS-Biotin in a buffer composed of 150 mM NaCl, 10 mM Triethanolamine, 2 mM CaCl_2_, and pH 7.5 for 20 min at 4 °C; and washed 3 times with PBS containing glycine (100 mM). Cells were lysed with a buffer composed of 1% Triton X-100, 150 mM NaCl, 5 mM EDTA, 50 mM Tris, pH 7.5, 1 mM NaVO_3_, 1 mM PMSF, and 1× protease inhibitor cocktail for 20 min at 4 °C and centrifuged at 14,000× *g* for 20 min at 4 °C. A total of 150–200 µg of protein was incubated with 100 μL of 50% slurry of Streptavidin-agarose beads (Pierce™ Streptavidin Agarose, #20349, Rockford, IL, USA) overnight at 4 °C. Beads were pelleted by centrifugation at 12,000 rpm for 2 min at 4 °C. Supernatants containing the non-biotinylated fraction representing intracellular proteins were collected. The beads were washed three times with lysis buffer, two times with high salt wash buffer (0.1% Triton X-100, 500 mM NaCl, 5 mM EDTA, 50 mM Tris pH: 7.5), and once with 10 mM Tris pH: 7.5. Biotinylated proteins representing membrane fraction were eluted by heating to 37 °C for 30 min or 95 °C for 5 min in 2× Laemmli sample buffer for detecting α1-NKA and β1-NKA, respectively. Equal amounts of non-biotinylated and biotinylated samples were used for SDS-PAGE and Western blotting. The success of cell surface biotinylation was considered by not detecting β-actin in surface (biotinylated) membrane fraction but only in the intracellular pool.

### 2.6. Preparation of Total Cell Lysates

For total protein expression studies, cells were washed three times with ice-cold PBS and lysed in a buffer composed of 1% Triton X-100, 150 mM NaCl, 5 mM EDTA, 50 mM Tris, pH 7.5, 1 mM NaVO_3_, 1 mM PMSF, and 1× protease inhibitor cocktail for 20 min at 4 °C and centrifuged at 14,000× *g* for 20 min at 4 °C. Supernatants containing total cellular proteins were frozen at −80 °C until use. Ten 30 μg of total cell lysates were used for SDS-PAGE and Western blotting to investigate the expression of α1-NKA and β1-NKA, respectively.

### 2.7. Western Blotting

For HIF-1α expression experiments, 20–30 μg of nuclear extracts were separated on 10% SDS-PAGE and transferred onto nitrocellulose membranes. Rabbit HIF-1α antibody (rabbit, #14179, Cell Signaling Technology, 1:1000 dilution) was used for Western blotting. For α1- and β1-NKA expression studies, mouse α1-NKA (ab7671, Abcam, Cambridge, UK, 1:2000 dilution) or rabbit β1-NKA antibody (Proteintech, UK, 15192-1-AP, 1:2000 dilution) were used. Beta-actin was used to normalize band densities as housekeeping (mouse, #A2228, Sigma, 1:10,000 dilution). Anti-mouse (GE Healthcare/Amersham, UK, #NA931, 1:2000–1:10,000 dilution) or anti-rabbit (#401315, Sigma, 1:2000) secondary antibodies conjugated with horseradish peroxidase and enhanced chemiluminescence (Amersham) were used for detection. Band densities were measured using the Image J 1.42q software (NIH, Bethesda, MD, USA).

### 2.8. NKA Activity Measurements

Ouabain sensitive ATPase activity was measured in cell membranes isolated from normoxic, hypoxic, or HIF-1α silenced H9c2 cells. Cells were scraped off the plates in ice-cold PBS and centrifuged at 1000× *g* for 5 min at 4 °C. Cell pellets were incubated for 30 min at 4 °C in 600 mM Sucrose and 10 mM Imidazole (pH 7.4) and passed through 27G needle. Crude homogenates were centrifugated at 2000× *g* for 10 min at 4 °C. Membrane pellets were obtained after centrifugation of the supernatants at 20,000× *g* for 30 min at 4 °C; they were suspended in a buffer composed of 250 mM Sucrose and 30 mM Imidazole (pH 7.4) and kept frozen at −80 °C until use.

For NKA activity measurements, 5–10 μg of plasma membrane proteins were incubated at 37 °C for 10 min in a buffer composed of (mM) NaCl 130, KCl 20, Tris 50 (pH 7.5), EGTA 1 (pH 7.5), NaN_3_ 5, MgCl_2_ 5, Ouabain 2, or DMSO as solvent control. Reaction was started by adding 5 mM Tris-ATP (pH 7.4) at a final concentration and incubated at 37 °C for 10 min. Ouabain-sensitive inorganic phosphate release was measured using the ammonium molybdate method as described previously [47] and calculated using linear regression with K_2_HPO_4_ as a standard. The difference between inorganic phosphate release in the absence or presence of ouabain indicated NKA activity. Values were normalized to protein amount used and presented as nmol/mg/mL.

### 2.9. Intracellular ATP Measurements

Total cellular ATP levels in normoxic, hypoxic, or HIF-1α silenced H9c2 cells were measured using Luminescent ATP Detection Assay Kit in 96 well plates according to the manufacturer’s suggestion (Abcam, ab113849, UK), and results were normalized to total protein levels.

### 2.10. Statistical Analysis

Results are shown as mean ± SD as indicated. Statistical analysis was performed using analysis of variance (ANOVA) for repeated measures and pair-wise multiple comparisons (LSD) or t-test as indicated using the SigmaPlot 10.0 (Systat Inc., Erkrath, Germany) software package. The level of statistical significance was *p* < 0.05.

## 3. Results

### 3.1. Silencing Efficiency of HIF-1α on the mRNA and Protein Expression of HIF-1α

**Figure 1:**
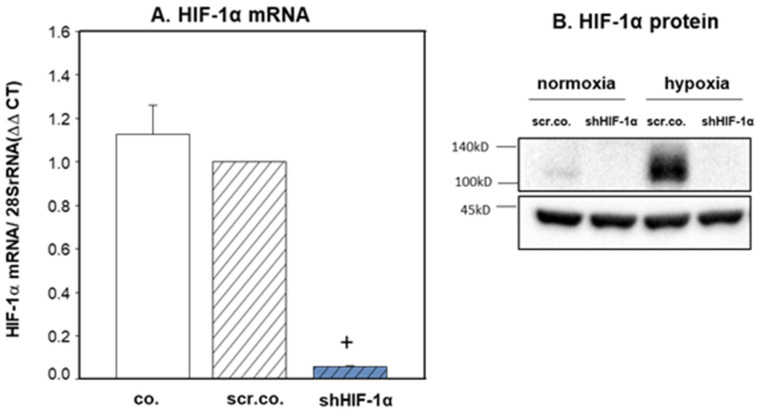
HIF-1α silencing on the expression of HIF-1α mRNA and protein. H9c2 cells were infected with 100 MOI scrambled or shHIF-1α containing adenoviral vectors and kept in normoxia (**A**) (19% O_2_) or hypoxia (**A**,**B**) (1% O_2_) for 24 h. mRNA expression was normalized to 28SrRNA. Mean values ± SD of 4 independent experiments normalized to normoxic scrambled control cells. The level of significance was *p* < 0.05: + effect of silencing compared to respective controls *p* < 0.001. co: non-infected cells, scr.co: scrambled control, shHIF-1α: HIF-1α silenced cells.

Silencing HIF-1α decreased the HIF-1α mRNA expression by more than 95% (*p* < 0.001) compared to the respective control cells (Figure 1A). Western blot experiments in nuclear extracts from normoxia, hypoxia, or HIF-1α silenced cells showed that HIF-1α was not detected in normoxia. Twenty-four hours of hypoxia increased the expression of HIF-1α, and it was totally downregulated in HIF-1α silenced cells (Figure 1B). These results indicate that the silencing efficiency of HIF-1α was ~95–100%.

### 3.2. Effect of Hypoxia and HIF-1α Silencing on the mRNA Expression of α1- and β1-NKA

**Figure 2:**
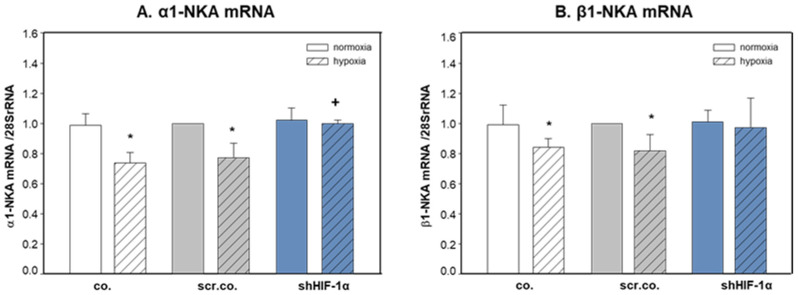
Effects of hypoxia and HIF-1α silencing on the mRNA expression of α1- and β1-NKA. H9c2 cells were infected with 100 MOI scrambled or shHIF-1α containing adenoviral vectors and kept in normoxia (19% O_2_) or hypoxia (1% O_2_) for 24 h. mRNA expression was normalized to 28SrRNA, and results are given as the normalized values of scr.co. Mean values ± SD of 4 independent experiments normalized to normoxic scr.co. The level of significance was *p* < 0.05: * effect of hypoxia compared to normoxia, + effect of HIF-1α silencing in hypoxic cells compared to hypoxic scr.co. co: non-infected cells, scr.co: scrambled virus control, shHIF-1α: HIF-1α silenced cells.

Figure 2A shows that hypoxia decreased the mRNA expression of α1-NKA by ~25% compared to normoxic cells (*p* = 0.015). Silencing HIF-1α totally prevented hypoxic inhibition on α1-NKA mRNA expression (*p* = 0.017). Hypoxia decreased β1-NKA mRNA expression slightly but significantly by ~15% compared to normoxic cells (*p* = 0.045). SilencingHIF-1α did not have any effect on β1-NKA mRNA expression (Figure 2B). These data indicate that hypoxia inhibits the mRNA expression of α1-NKA and β1-NKA, but only mRNA expression of α1-NKA in H9c2 cells is controlled by HIF-1α.

### 3.3. Effect of Hypoxia and HIF-1α Silencing on α1-NKA and β1-NKA Membrane, Intracellular, and Total Expression

**Figure 3:**
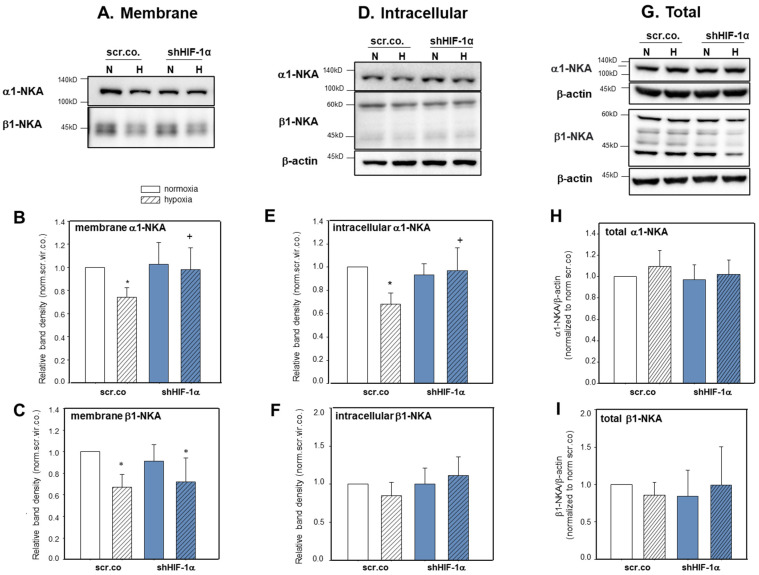
Effect of hypoxia and HIF-1α silencing on the abundance of α1-NKA and β1-NKA in plasma membranes, cytoplasmic fractions, and total levels of H9c2 cells. H9c2 cells were silenced with scrambled or shHIF-1α containing adenoviral vectors and kept in normoxia (19% O_2_) or hypoxia (1% O_2_). Biotinylated fraction is considered plasma membrane proteins; non-biotinylated is intracellular proteins. Representative blot showing α1-NKA (**A**,**B**) and β1-NKA (**A**,**C**) surface expression (biotinylated) and intracellular (non-biotinylated) expression (**D**–**F**). Representative blot showing total protein levels of α1-NKA (**G**,**H**) and β1-NKA (**G**,**I**). The lack of β -actin expression in the biotinylated fractions indicates the purity of the membrane pull-down. Mean values ± SD of 5–6 independent experiments normalized to normoxic scr.co. The level of significance was *p* < 0.05: * effect of hypoxia compared to normoxia, + effect of HIF-1α silencing in hypoxic cells compared to hypoxic scr.co. scr.co: scrambled control, shHIF-1α: HIF-1α silenced cells.

We tested next whether hypoxia and HIF-1α have any influence on the plasma membrane expression and intracellular trafficking of α1-NKA and β1-NKA proteins. Figure 3A,B show that the plasma membrane abundance of α1-NKA decreased by ~27% in hypoxic cells compared to normoxic controls (*p* = 0.038). Silencing HIF-1α fully prevented this effect (*p* = 0.047; hypoxia scr.co. v.s. hypoxia HIF-1α silencing). Hypoxia decreased the plasma membrane abundance of β1-NKA protein compared to normoxic cells by ~35% (Figure 3A,C, *p* = 0.001). There was no effect of HIF-1α silencing (*p* = 0.971). Non-biotinylated fractions represent the intracellular expression of the proteins. Figure 3D,E show decreased cytosolic protein levels of α1-NKA in hypoxic cells compared to normoxic controls (*p* = 0.029), which was prevented by silencing HIF-1α (*p* = 0.041). The intracellular expression of β1-NKA did not change by hypoxia or HIF-1α silencing. Figure 3G–I show that total protein levels of α1-NKA and β1-NKA were not affected by hypoxia or HIF-1α silencing. These data indicate that hypoxia caused internalization of α1-NKA from the plasma membrane, and it is controlled by HIF-1α dependent mechanisms (Figure 3E). The decrease in the plasma membrane abundance of β1-NKA in hypoxia suggests internalization and degradation (Figure 3F). Together, these data show that hypoxia and HIF-1α control the intracellular trafficking of α1- and β1-NKA differently.

### 3.4. Effect of Hypoxia and HIF-1α Silencing on NKA Activity and Total ATP Levels

**Figure 4:**
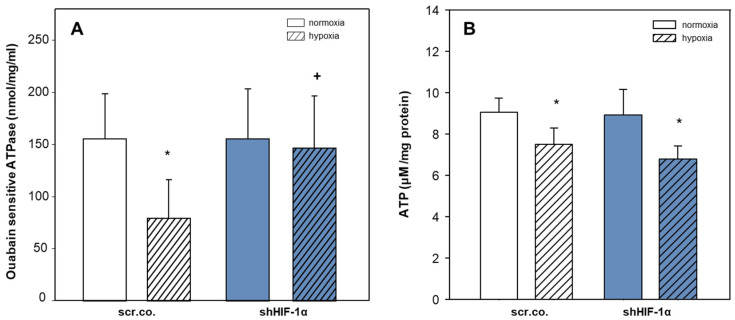
Effect of hypoxia and HIF-1α silencing on the activity of NKA and total ATP levels. Plasma membranes were isolated from scrambled or shHIF-1α silenced H9c2 cells that were kept in normoxia (19% O_2_) or hypoxia (1% O_2_) for 24 h. Ouabain-sensitive ATPase activity indicates NKA activity (**A**). Mean values ± SD of 6 independent membrane preparations. One-way ANOVA; the level of significance was *p* < 0.05. Total ATP levels were measured by luciferase-luciferin assay, and values were normalized to total protein (**B**). Mean values ± SD of 4 independent experiments performed in triplicates. The level of significance was *p* < 0.05: * effect of hypoxia compared to normoxia, + effect of HIF-1α silencing in hypoxic cells compared to hypoxic scr.co. scr.co: scrambled control, shHIF-1α: HIF-1α silenced cells.

We also measured the activity of NKA and total ATP levels to interpret any changes that might have occurred in the pump activity. Figure 4A shows that hypoxia decreased ouabain-sensitive ATPase activity by ~50% compared to normoxia (*p* = 0.008) and that silencing HIF-1α fully prevented hypoxic inhibition (*p* = 0.018; hypoxia scr.co. v.s. hypoxia HIF-1α silencing). Cellular ATP levels decreased by hypoxia compared to normoxia by ~17% (*p* = 0.024, Figure 4B). Silencing HIF-1α in hypoxic cells did not further affect total ATP levels (*p* = 0.209). These data indicate that the hypoxic inhibition of NKA activity in H9c2 cardiomyoblasts is HIF-1α dependent, and the decreased levels of ATP in hypoxic HIF-1α silenced cells do not limit the functionality of NKA. Moreover, the restored activity of NKA in HIF-1α silenced cells does not further decline ATP levels (Figure 4A,B).

## 4. Discussion

This study is the first to show that the 24 h hypoxia exposure of H9c2 cardiomyoblasts decreases plasma membrane abundance and increases the internalization of α1-NKA. Hypoxia-mediated effects were controlled by HIF-1α because silencing HIF-1α prevented internalization and, thus, increased insertion of the proteins into the plasma membrane. Moreover, the decreased activity of NKA in hypoxic H9c2 cardiomyoblasts was HIF-1α dependent, and declined intracellular ATP level was not limiting to the functionality of NKA. Our results also showed that the decreased membrane expression of β1-NKA by hypoxia was due to increased internalization and degradation of the protein.

The function and regulation of NKA in cardiovascular diseases (CVD) has been extensively studied with human tissue samples from end-stage cardiac-disease patients and various animal models using different experimental approaches. The decreased activity of NKA has been mainly associated with the decreased expression of the isoforms. However, discrepant findings on expression studies do not explain the activity of NKA and lack mechanisms to clarify the observed effects [23]. Given that tissue hypoxia is a common feature and clinical finding in CVD, we hypothesized that HIF-1α might be involved in regulating the expression of NKA isoforms and activity and that the observed inconsistent findings might be due to varying degrees of the hypoxic stress the tissues were exposed to. To achieve the conditions of comparable degrees of hypoxia and model-related cardiac injury, we generated an in-vitro model of hypoxic myocardium using H9c2 rat ventricular cardiomyoblasts, one of the most used cell lines in studies on cardio-myocyte cellular functions, and aimed to provide a mechanism of regulation of α1-and β1-NKA and activity of NKA in the hypoxic heart.

Our result showed that hypoxia decreased mRNA expressions of α1-NKA and β1-NKA by about 25% and 15%, respectively. The decreased mRNA expression of α1-NKA was prevented by silencing HIF-1α, indicating its involvement in the regulation. The slight but significant decrease in the mRNA expression of β1-NKA was not dependent on HIF-1α. Hypoxia inhibits the transcription and translation of some proteins to adapt and allow cells to consume less ATP for the maintenance of their viability and survival. Under hypoxic conditions, HIFs play critical roles for the adjustment of cells to low oxygen levels [48]. Here, we showed that silencing HIF-1α did not further affect hypoxic inhibition on ATP levels, which is in agreement with previous reports [49,50]. Despite the decrease in mRNA levels by hypoxia, the total protein expression of α1-NKA and β1-NKA did not change, indicating that the transcriptional changes occurred by hypoxia did not affect translation into protein levels. Also, the prevention of the decreased mRNA expression of α1-NKA by HIF-1α in hypoxic cells did not affect total protein expression. These data show that despite the changes in the transcription of α1-NKA and β1-NKA, the efficiency of mRNA translation can still be maintained in hypoxia. Therefore, the observed decrease in the membrane abundance of the α1-NKA and β1-NKA subunits were not due to decreased total protein levels.

Majority of the studies focused on the expression of NKA isoforms in tissues or cells have used total homogenates or crude membrane fractions, thus providing limited information. Therefore, the plasma membrane expression of the NKA subunits need to be accurately measured where the active pump subunits are expressed. In this study, we used the cell-impermeable Sulfo-NHS-SS-Biotin that binds to extracellular sites of the proteins and pulled-down biotin-bound proteins; non-biotinylated fraction indicated intracellular protein pool. Here, we report for the first time that the membrane abundance of α1- and β1-NKA decreased in hypoxic H9c2 cardiomyoblasts. Hypoxia decreased the intracellular expression of α1-NKA, indicating the internalization of the protein from the plasma membrane. HIF-1α silencing in hypoxic cells prevented this effect; thus, it increased membrane insertion. Hypoxia also decreased the plasma membrane abundance of β1-NKA, whereas intracellular expression was not affected, suggesting that membrane β1-NKA was internalized into the cell and degraded. This effect was independent of HIF-1α.

We also showed that the hypoxic inhibition of NKA activity depends on increased HIF-1α, and decreased cellular ATP is not a limiting factor for restoring decreased pump activity. The mechanism behind this finding is not known. One possible explanation for the observed effects might be the increased insertion of α1-NKA into the plasma membrane in hypoxic HIF-1α silenced cells. In these conditions, the pool of β1-NKA might still be enough to contribute to the functionality of the pump despite decreased expression. Using an in-vitro model of acute ischemia reperfusion (I/R) injury, Belliard et al. demonstrated that internalization of α1-NKA was prevented by ouabain preconditioning in a PKCε-dependent manner. However, decreased NKA activity in I/R was not reversed by the inhibition of PKCε [51]. It may be likely that different signaling pathways regulate the expression and activity of NKA in an acute I/R model. In our study, the long-term effect of hypoxia on the expression of α1-NKA and activity of NKA in H9c2 cells was HIF-1α dependent. The signaling molecules mediating the observed effects by HIF-1α remain to be determined.

The decreased activity of NKA in hypoxia was also linked to redox dependent changes in the cells, such as the increased oxidized-glutathione-dependent modification of the thiols in the cysteine residue of α1-NKA [52]. Petrushanko et al. reported that in hypoxic rat myocardium, the S-glutathionylation of the α1-subunit was associated with oxidative stress, ATP depletion, and decreased NKA activity [53]. In contrast, Kurella et al. reported that even if the thiol groups of NKA were fully oxidized, it would not be enough to explain the impaired pump activity [54]. Figtree et al. and Bibert et al. reported that β1-NKA also undergoes to redox-dependent modifications, causing an inhibition of the pump activity [55,56]. Given that during prolonged hypoxia, oxidative enzyme capacities, NO, ROS, and glutathione levels decrease, additional changes that occur might contribute to the decreased activity of NKA. The mechanism behind the reversal of blunted NKA activity in hypoxic HIF-1α silenced cells requires further investigation.

The hypoxia-HIF-dependent modulation of the activity and expression of some membrane receptors, ion channels, and transporters has been reported in different cells and tissues. In hypoxic cells, the plasma membrane expression of the beta2 adrenergic receptor decreased due to the ubiquitylation of the receptor by von Hippel–Lindau tumor suppressor protein (pVHL)-E3 ligase complex, which regulates HIF-1α expression [57]. In breast cancer cells and in HL-1 cardiomyocytes, hypoxia increased the expression and activity of G protein-coupled receptor 30 (GPR30) in HIF-1α-dependent manner [58]. In lung alveolar epithelial cells, severe hypoxia caused stabilization of the plasma membrane expression of α1-NKA by HIF-dependent protein kinase C zeta (PKC ζ) degradation [59]. We recently showed HIF-2α mediated internalization and degradation of plasma membrane expression of epithelial Na channels (ENaC) in primary alveolar epithelial cells exposed to in-vitro hypoxia. We also showed that the instillation of shHIF-2α expressing adenoviral vector prior to hypoxia exposure improved the lung fluid reabsorption and decreased in in-vivo hypoxia [45]. Recent studies have reported the regulation of calcium and voltage-activated potassium channels (BKs) by hypoxia, as well as HIF-1α and its relation to CVD [60]. Further experiments are needed to investigate whether HIF mediated regulations of membrane proteins are restricted to some classes.

Our study has limitations. The results obtained from this study need to also be tested on the cellular level in an in-vivo model of ischemic heart disease because the H9c2 cardiomyoblast cell line used in this study does not fully match the phenotype and functions of mature cardiomyocytes.

## 5. Conclusions

Taken together, our results showed for the first time that 24 h hypoxia of H9c2 cardiomyoblasts decreased the plasma membrane expression of α1-and β1-NKA and activity of NKA. Under hypoxic conditions, α1-NKA was internalized, and HIF-1α was involved in this regulation. The β1-NKA was internalized and degraded independently of HIF-1α. The decreased activity of NKA in hypoxic H9c2 cardiomyoblasts was also regulated by HIF-1α. Moreover, although cellular ATP decreased, the levels were still enough to drive the NKA activity in hypoxia and even in the absence of HIF-1α. The mechanism behind this regulation on the molecular basis and any effects on other Na^+^ and Ca^2+^ transporting systems need further investigation.

## Data Availability

Not applicable.

## References

[1] Skou J.C. (1957). The influence of some cations on an adenosine triphosphatase from peripheral nerves. Biochim. Biophys. Acta.

[2] Geering K. (2006). Fxyd proteins: New regulators of Na-K-atpase. Am. J. Physiol. Renal Physiol..

[3] Yan Y., Shapiro J.I. (2016). The physiological and clinical importance of sodium potassium atpase in cardiovascular diseases. Curr. Opin. Pharmacol..

[4] Xie Z.J., Xie J. (2005). The Na/K-atpase-mediated signal transduction as a target for new drug development. Front. Biosci-Landmrk.

[5] Bouzinova E.V., Hangaard L., Staehr C., Mazur A., Ferreira A., Chibalin A.V., Sandow S.L., Xie Z., Aalkjaer C., Matchkov V.V. (2018). The alpha2 isoform Na,K-atpase modulates contraction of rat mesenteric small artery via csrc-dependent Ca(2+) sensitization. Acta Physiol..

[6] Peng M., Huang L., Xie Z., Huang W.H., Askari A. (1996). Partial inhibition of Na^+^/K^+^-atpase by ouabain induces the Ca^2+^-dependent expressions of early-response genes in cardiac myocytes. J. Biol. Chem..

[7] Xie Z., Askari A. (2002). Na(+)/K(+)-atpase as a signal transducer. Eur. J. Biochem..

[8] Haas M., Askari A., Xie Z. (2000). Involvement of src and epidermal growth factor receptor in the signal-transducing function of Na^+^/K^+^-atpase. J. Biol. Chem..

[9] Liu J., Tian J., Haas M., Shapiro J.I., Askari A., Xie Z. (2000). Ouabain interaction with cardiac Na^+^/K^+^-atpase initiates signal cascades independent of changes in intracellular Na^+^ and Ca^2+^ concentrations. J. Biol. Chem..

[10] Kometiani P., Li J., Gnudi L., Kahn B.B., Askari A., Xie Z. (1998). Multiple signal transduction pathways link Na^+^/K^+^-atpase to growth-related genes in cardiac myocytes. The roles of ras and mitogen-activated protein kinases. J. Biol. Chem..

[11] Aydemir-Koksoy A., Abramowitz J., Allen J.C. (2001). Ouabain-induced signaling and vascular smooth muscle cell proliferation. J. Biol. Chem..

[12] Tian J., Liu J., Garlid K.D., Shapiro J.I., Xie Z. (2003). Involvement of mitogen-activated protein kinases and reactive oxygen species in the inotropic action of ouabain on cardiac myocytes. A potential role for mitochondrial k(atp) channels. Mol. Cell Biochem..

[13] Tian J., Cai T., Yuan Z., Wang H., Liu L., Haas M., Maksimova E., Huang X.Y., Xie Z.J. (2006). Binding of src to Na^+^/K^+^-atpase forms a functional signaling complex. Mol. Biol. Cell.

[14] Morth J.P., Pedersen B.P., Toustrup-Jensen M.S., Sorensen T.L., Petersen J., Andersen J.P., Vilsen B., Nissen P. (2007). Crystal structure of the sodium-potassium pump. Nature.

[15] Blanco G., Mercer R.W. (1998). Isozymes of the Na-K-atpase: Heterogeneity in structure, diversity in function. Am. J. Physiol..

[16] Jimenez T., McDermott J.P., Sanchez G., Blanco G. (2011). Na,K-atpase alpha4 isoform is essential for sperm fertility. Proc. Natl. Acad. Sci. USA.

[17] Berry R.G., Despa S., Fuller W., Bers D.M., Shattock M.J. (2007). Differential distribution and regulation of mouse cardiac Na^+^/K^+^-atpase alpha1 and alpha2 subunits in t-tubule and surface sarcolemmal membranes. Cardiovasc. Res..

[18] Sweadner K.J., Herrera V.L., Amato S., Moellmann A., Gibbons D.K., Repke K.R. (1994). Immunologic identification of Na^+^,K(+)-atpase isoforms in myocardium. Isoform change in deoxycorticosterone acetate-salt hypertension. Circ. Res..

[19] Aronsen J.M., Swift F., Sejersted O.M. (2013). Cardiac sodium transport and excitation-contraction coupling. J. Mol. Cell Cardiol..

[20] Chow D.C., Forte J.G. (1995). Functional significance of the beta-subunit for heterodimeric p-type atpases. J. Exp. Biol..

[21] Geering K. (2008). Functional roles of Na,K-atpase subunits. Curr. Opin. Nephrol. Hypertens..

[22] Shattock M.J., Ottolia M., Bers D.M., Blaustein M.P., Boguslavskyi A., Bossuyt J., Bridge J.H., Chen-Izu Y., Clancy C.E., Edwards A. (2015). Na^+^/Ca^2+^ exchange and Na^+^/K^+^-atpase in the heart. J. Physiol..

[23] Baloglu E. (2023). Hypoxic stress-dependent regulation of Na,K-atpase in ischemic heart disease. Int. J. Mol. Sci..

[24] Semenza G.L. (2014). Hypoxia-inducible factor 1 and cardiovascular disease. Annu. Rev. Physiol..

[25] Lee S.H., Wolf P.L., Escudero R., Deutsch R., Jamieson S.W., Thistlethwaite P.A. (2000). Early expression of angiogenesis factors in acute myocardial ischemia and infarction. N. Engl. J. Med..

[26] Jurgensen J.S., Rosenberger C., Wiesener M.S., Warnecke C., Horstrup J.H., Grafe M., Philipp S., Griethe W., Maxwell P.H., Frei U. (2004). Persistent induction of hif-1alpha and -2alpha in cardiomyocytes and stromal cells of ischemic myocardium. FASEB J..

[27] Sui X., Wei H., Wang D. (2015). Novel mechanism of cardiac protection by valsartan: Synergetic roles of tgf-beta1 and hif-1alpha in ang ii-mediated fibrosis after myocardial infarction. J. Cell Mol. Med..

[28] Krishnan J., Suter M., Windak R., Krebs T., Felley A., Montessuit C., Tokarska-Schlattner M., Aasum E., Bogdanova A., Perriard E. (2009). Activation of a hif1alpha-ppargamma axis underlies the integration of glycolytic and lipid anabolic pathways in pathologic cardiac hypertrophy. Cell Metab..

[29] Abe H., Takeda N., Isagawa T., Semba H., Nishimura S., Morioka M.S., Nakagama Y., Sato T., Soma K., Koyama K. (2019). Macrophage hypoxia signaling regulates cardiac fibrosis via oncostatin m. Nat. Commun..

[30] Tang H., Babicheva A., McDermott K.M., Gu Y., Ayon R.J., Song S., Wang Z., Gupta A., Zhou T., Sun X. (2018). Endothelial hif-2alpha contributes to severe pulmonary hypertension due to endothelial-to-mesenchymal transition. Am. J. Physiol. Lung Cell Mol. Physiol..

[31] Bogdanova A., Ogunshola O.O., Bauer C., Nikinmaa M., Gassmann M. (2003). Molecular mechanisms of oxygen-induced regulation of Na^+^/K^+^ pump. Adv. Exp. Med. Biol..

[32] Schwinger R.H., Wang J., Frank K., Muller-Ehmsen J., Brixius K., McDonough A.A., Erdmann E. (1999). Reduced sodium pump alpha1, alpha3, and beta1-isoform protein levels and Na^+^,K^+^-atpase activity but unchanged Na^+^-Ca^2+^ exchanger protein levels in human heart failure. Circulation.

[33] Semb S.O., Lunde P.K., Holt E., Tonnessen T., Christensen G., Sejersted O.M. (1998). Reduced myocardial Na^+^, K(+)-pump capacity in congestive heart failure following myocardial infarction in rats. J. Mol. Cell Cardiol..

[34] Bossuyt J., Ai X., Moorman J.R., Pogwizd S.M., Bers D.M. (2005). Expression and phosphorylation of the na-pump regulatory subunit phospholemman in heart failure. Circ. Res..

[35] Ishino K., Botker H.E., Clausen T., Hetzer R., Sehested J. (1999). Myocardial adenine nucleotides, glycogen, and Na, K-atpase in patients with idiopathic dilated cardiomyopathy requiring mechanical circulatory support. Am. J. Cardiol..

[36] Pike M.M., Luo C.S., Clark M.D., Kirk K.A., Kitakaze M., Madden M.C., Cragoe E.J., Pohost G.M. (1993). Nmr measurements of Na^+^ and cellular energy in ischemic rat heart: Role of Na(+)-H^+^ exchange. Am. J. Physiol..

[37] Verdonck F., Volders P.G., Vos M.A., Sipido K.R. (2003). Intracellular Na^+^ and altered Na^+^ transport mechanisms in cardiac hypertrophy and failure. J. Mol. Cell Cardiol..

[38] Despa S., Islam M.A., Weber C.R., Pogwizd S.M., Bers D.M. (2002). Intracellular Na(+) concentration is elevated in heart failure but Na/K pump function is unchanged. Circulation.

[39] MacLeod K.T. (2023). Changes in cellular Ca(2+) and Na(+) regulation during the progression towards heart failure. J. Physiol..

[40] Swift F., Birkeland J.A., Tovsrud N., Enger U.H., Aronsen J.M., Louch W.E., Sjaastad I., Sejersted O.M. (2008). Altered Na^+^/Ca^2+^-exchanger activity due to downregulation of Na^+^/K^+^-atpase alpha2-isoform in heart failure. Cardiovasc. Res..

[41] Shamraj O.I., Grupp I.L., Grupp G., Melvin D., Gradoux N., Kremers W., Lingrel J.B., De Pover A. (1993). Characterisation of Na/K-atpase, its isoforms, and the inotropic response to ouabain in isolated failing human hearts. Cardiovasc. Res..

[42] Jager H., Wozniak G., Akinturk I.H., Hehrlein F.W., Scheiner-Bobis G. (2001). Expression of sodium pump isoforms and other sodium or calcium ion transporters in the heart of hypertensive patients. Biochim. Biophys. Acta.

[43] Fedorova O.V., Talan M.I., Agalakova N.I., Lakatta E.G., Bagrov A.Y. (2004). Coordinated shifts in Na/K-atpase isoforms and their endogenous ligands during cardiac hypertrophy and failure in nacl-sensitive hypertension. J. Hypertens..

[44] Book C.B., Moore R.L., Semanchik A., Ng Y.C. (1994). Cardiac hypertrophy alters expression of Na^+^,K(+)-atpase subunit isoforms at mrna and protein levels in rat myocardium. J. Mol. Cell Cardiol..

[45] Baloglu E., Nonnenmacher G., Seleninova A., Berg L., Velineni K., Ermis-Kaya E., Mairbaurl H. (2020). The role of hypoxia-induced modulation of alveolar epithelial Na(+)- transport in hypoxemia at high altitude. Pulm. Circ..

[46] Rao X., Huang X., Zhou Z., Lin X. (2013). An improvement of the 2^(-delta delta ct) method for quantitative real-time polymerase chain reaction data analysis. Biostat. Bioinforma Biomath..

[47] Forbush B. (1983). Assay of Na,K-atpase in plasma membrane preparations: Increasing the permeability of membrane vesicles using sodium dodecyl sulfate buffered with bovine serum albumin. Anal. Biochem..

[48] Sato T., Takeda N. (2023). The roles of hif-1alpha signaling in cardiovascular diseases. J. Cardiol..

[49] Loeh B., Baloglu E., Ke A., Bartsch P., Mairbaurl H. (2010). Beta2-adrenergic stimulation blunts inhibition of epithelial ion transport by hypoxia of rat alveolar epithelial cells. Cell Physiol. Biochem..

[50] Wodopia R., Ko H.S., Billian J., Wiesner R., Bartsch P., Mairbaurl H. (2000). Hypoxia decreases proteins involved in epithelial electrolyte transport in a549 cells and rat lung. Am. J. Physiol. Lung Cell Mol. Physiol..

[51] Belliard A., Sottejeau Y., Duan Q., Karabin J.L., Pierre S.V. (2013). Modulation of cardiac Na^+^,K^+^-atpase cell surface abundance by simulated ischemia-reperfusion and ouabain preconditioning. Am. J. Physiol. Heart Circ. Physiol..

[52] Petrushanko I.Y., Mitkevich V., Makarov A. (2020). Molecular mechanisms of the redox regulation of the Na, K-atpase. Biophysics.

[53] Petrushanko I.Y., Yakushev S., Mitkevich V.A., Kamanina Y.V., Ziganshin R.H., Meng X., Anashkina A.A., Makhro A., Lopina O.D., Gassmann M. (2012). S-glutathionylation of the Na,K-atpase catalytic alpha subunit is a determinant of the enzyme redox sensitivity. J. Biol. Chem..

[54] Kurella E.G., Tyulina O.V., Boldyrev A.A. (1999). Oxidative resistance of Na/K-atpase. Cell Mol. Neurobiol..

[55] Figtree G.A., Liu C.C., Bibert S., Hamilton E.J., Garcia A., White C.N., Chia K.K., Cornelius F., Geering K., Rasmussen H.H. (2009). Reversible oxidative modification: A key mechanism of Na^+^-K^+^ pump regulation. Circ. Res..

[56] Bibert S., Liu C.C., Figtree G.A., Garcia A., Hamilton E.J., Marassi F.M., Sweadner K.J., Cornelius F., Geering K., Rasmussen H.H. (2011). Fxyd proteins reverse inhibition of the Na^+^-K^+^ pump mediated by glutathionylation of its beta1 subunit. J. Biol. Chem..

[57] Xie L., Xiao K., Whalen E.J., Forrester M.T., Freeman R.S., Fong G., Gygi S.P., Lefkowitz R.J., Stamler J.S. (2009). Oxygen-regulated beta(2)-adrenergic receptor hydroxylation by egln3 and ubiquitylation by pvhl. Sci. Signal.

[58] Recchia A.G., De Francesco E.M., Vivacqua A., Sisci D., Panno M.L., Ando S., Maggiolini M. (2011). The g protein-coupled receptor 30 is up-regulated by hypoxia-inducible factor-1alpha (hif-1alpha) in breast cancer cells and cardiomyocytes. J. Biol. Chem..

[59] Magnani N.D., Dada L.A., Queisser M.A., Brazee P.L., Welch L.C., Anekalla K.R., Zhou G., Vagin O., Misharin A.V., Budinger G.R.S. (2017). Hif and hoil-1l-mediated pkczeta degradation stabilizes plasma membrane Na,K-atpase to protect against hypoxia-induced lung injury. Proc. Natl. Acad. Sci. USA.

[60] Ochoa S.V., Otero L., Aristizabal-Pachon A.F., Hinostroza F., Carvacho I., Torres Y.P. (2021). Hypoxic regulation of the large-conductance, calcium and voltage-activated potassium channel, bk. Front. Physiol..

